# Dissertation on the Diseases of the Maxillary Sinus

**Published:** 1842-09

**Authors:** Chapin A. Harris

**Affiliations:** Professor of Practical Dentistry in the Baltimore College of Dental Surgery, &c.


### ARTICLE II.

*Dissertation on the Diseases of the Maxillary Sinus.* Read before the American Society of Dental Surgeons, at their third annual meeting; held in the city of Boston, July 20th, 1842. By CHAPIN A. HARRIS, M. D., D. D. S., Professor of Practical Dentistry in the Baltimore College of Dental Surgery, &c.

*Mr. President and Gentlemen:*

AT the last meeting of this association, I had the honour to submit to your consideration, a memoir on the "Characteristics of the teeth, gums, &c. &c." and I now beg leave to submit another, on the diseases of the maxillary sinus, and in doing this, I am actuated by the belief, that a short, and at the same time, comprehensive treatise, on the morbid affections of this cavity, would not be altogether unacceptable to the members of the dental profession.

Few works, devoted exclusively to these diseases, and none in our own language, that I am aware of, has ever been published. Most that has been written upon them is contained in works on general surgery; many of which are either out of print, or can be obtained only with great difficulty. Mr. Thomas Bell, it is true, in his excellent work, entitled, "The Anatomy, Physiology, and Diseases of the Teeth," treats upon them at considerable length; but, it could hardly be expected that, in a publication of this kind, as much space should be given to the diseases of this cavity, as their importance demands. The description given of them, however, by this able and accomplished writer, discovers an accuracy of observation that could only be possessed by such as have made them a subject of close and thorough investigation. I might also mention the names of others, who have written upon one or more of these affections, but it is unnecessary to do so now; I may hereafter have occasion to refer to some of their opinions.

But, although the diseases of the maxillary sinus, have engaged the attention of many able writers, little, comparatively, of what has been written upon them, is accessible to the members generally, of the dental profession; and in view of this fact, I have been induced to attempt the preparation of a brief treatise upon them. That one is needed, all, I think, will unhesitatingly admit; and that is is important that the dental practitioner should understand the pathology and curative indications of these affections, none, I believe, will deny; for, caused as they in most instances are, by an unhealthy condition of the teeth, their treatment, as is remarked by Mr. Bell, "is so intimately connected with that of these organs, as to be universally considered as forming a part of the legitimate object of this branch of practice."

With these prefatory remarks, I shall proceed, firstly, to make a few general observations on the morbid affections of this cavity, and afterwards to notice separately each of the diseases to which it is liable.

*Diseases of the Maxillary Sinus.*

In the remarks which I am about to offer on the diseases of the maxillary sinus, it will be unnecessary to give a full description of this cavity, inasmuch as that has been done, by nearly every writer on general anatomy, since the middle of the seventeenth century. It will be sufficient to observe, that it is of an irregular quadrangular shape, and situated in the middle of each superior maxillary bone, between the orbiter plate and the palatine and alveolar processes, that it is lined by the pituitary membrane, and communicates with the nose by means of an opening between the superior and inferior turbinated bones, which, though large in the skeleton, is not more than one-fourth or fifth the size of a common goose-quill in the living subject, and is always partially closed by a duplicature or fold of the mucous membrane.*

It was not however, until the knowledge of anatomy had made considerable progress, that the existence of this cavity was known, *Casserius*, an anatomist of *Padua*, who flourished during the latter part of the sixteenth and early part of the seventeenth centuries, is said to have been the first to discover it; but no correct description of it was given, until about the middle of the latter, and to *Nathaniel Highmore*, author of a treatise on anatomy,† published in 1651, the credit of this belongs. Hence the name of "*antrum highmorianum,*" by which it is usually designated.

This cavity is subject to some of the most formidable and dangerous forms of disease that the medical or surgical practitioner is ever called upon to treat; and yet, there are few diseases

* Vide Memoires de l'Academie de Chirurgie. Tom. 12, edit. in 12, p. 4.

† This work is entitled, "*Corporis humani Desquisitio Anitomico.*"

incident to the human body, that have not received more attention from writers on pathology and therapeutics than these. Diseases are sometimes here met with, over which neither the surgeon nor physician can exercise any control, and whose progress is only arrested with that of the life of the unfortunate sufferer.

All of the diseases to which the antrum maxillare is subject, however, are not of so dangerous a character; some are very simple and easily cured, but even those which are regarded as the least dangerous, and that yield most readily to treatment, when instituted during their incipient or early stages, often, if neglected for a considerable length of time, or if improperly treated, assume a new and so aggravated a form, as to bid defiance alike to the skill both of the physician and surgeon. But, while on the one hand, the most simple affections that are here met with, may by neglect or improper treatment, ultimately become incurable; those on the other, which are considered as the most malignant and dangerous from their inception, might, I have no doubt, by a timely and judicious employment of suitable remedies, be effectually and radically removed.

The form which the disease puts on, it must be admitted, is determined by the state of the constitutional health or some specific tendency of the general system, and we can therefore readily imagine, that a cause which in one person, would give rise only to simple inflammation of the lining membrane, or a mucous engorgement of the sinus, would in another, produce an ill conditioned ulcer, fungus hæmatodes or asteo-sarcoma. But simple inflammation and mucous engorgement of this cavity, not unfrequently cause caries and exfoliations of its surrounding osseous tissues, and as a consequence in some instances, even the destruction of the life of the patient.

The importance then of early attention to the diseases of this cavity, is very apparent, and this is the more necessary as it is often difficult and sometimes even impossible to determine the character of the malady, until it has progressed so far as to have involved, to a greater or less extent, the neighbouring parts; when, if it has not become incurable, its removal is, at least, rendered more tedious and less easy of accomplishment. It may therefore, be safely assumed, that in a very large majority of the cases of disease of the maxillary sinus, the danger to be apprehended, results more from neglect, than any necessarily fatal character of the malady, so that in forming a prognosis, the circumstances to be considered, should be, the state of the constitutional health, the progress made by the affection, and the nature and extent of the injury inflicted by it upon the surrounding tissues. If the general health be not so much impaired as to prevent its restoration by the employment of proper remedies, and the disease has not inflicted extensive and irreparable injury upon the neighbouring structures, the prognosis will be favourable; but if the functional operations of the body have become so much deranged as to prevent their restoration, and the bones of the face and nose have become seriously implicated with the affection of the sinus, the combined resources both of medicine and surgery will prove unavailing.

In young and middle aged subjects of good constitutions, a morbid action may exist in the antrum for years without giving rise to any very alarming symptoms, while the same species of affection in another less healthy constituted, would rapidly extend itself and degenerate into so malignant a form of disease as to threaten the speedy destruction of the life of the patient. Medical history abounds with examples of this kind, and they conclusively establish, that the state of the general health and habit of body, whatever may have been the primitive characteristics of the malady, ultimately determine its malignancy, and that in the treatment of affections of this cavity, as well as other local diseases of the body, these should not be overlooked.

But, independently of the danger of the affections which seat themselves here, they, for the most part are very loathsome, and subject the patient to great annoyance. They change the qualities of the secretions of this cavity and cause them to exhale a very nauseating and fetid odor. This, in many instances, is almost insufferable to the patient, and when it is considered that these secretions must have egress through some opening, and when prevented from escaping through the natural one into the nose, as is often the case, that they evacuate themselves through an artificial one formed by art, or effected by their own acrimonious and disorganizing qualities, through the cheek, alveolar border or palatine arch, the inconvenience, it can readily be imagined, must be great. And, they are not only exceedingly annoying, but they are also sometimes very painful; but the degree and constancy of the pain by which they are attended, vary according to their nature and the nervous susceptibility of the individual. The pain is sometimes lancinating and excruciating almost beyond the power of endurance, but when it is very severe, it is usually less constant; at other times it is slight, but more constant.

The occurrence of disease in the maxillary sinus, as I have already intimated, is often very insiduous. It not unfrequently happens that it exists for weeks and even months before its presence is suspected—the slight uneasiness occasioned by it being attributed to some morbid condition of the teeth or gums, and the symptoms attendant upon one description of affection of this cavity, are often so similar to those that accompany another, that it is impossible to determine the character of disease that is developing itself, until it has made considerable progress.

But man is not the only animal that is subject to morbid affections of the antrum maxillare. Dr. Wm. Cook,* states that while residing in the country, a disease prevailed among cattle which caused them to go mad, and that it was believed that they had been bitten by a mad dog. Having a desire to ascertain the seat and nature of the malady, which was attended with such singular, and in most instances, fatal effects, he examined the head of one that had died of it. His researches were first directed to the brain, but as no morbid appearances were there observable, he next laid open the superior maxillary, and to his great astonishment, found the antrum filled with fetid matter. But although he was fortunate enough to discover the seat of the disease, he does not attempt to give any explanation of its cause, nor does he even state the season of the year at which it prevailed. The affection however, evidently consisted of a mucous engorgement of the sinus, accompanied, most probably, by ulceration of its lining membrane and caries of its osseous walls.

The morbid affections of the maxillary sinus, are, for the most part, similar to those of the nasal fassæ. There is, however, one

* Vide note to Morgagni's work on the Seats and Causes of Diseases, &c. &c. vol. i. page 167.

form of disease which seems to be peculiar to this cavity, and that is mucous engorgement. Deschamps mentions two, dropsy and purulent accumulations,* but the first of these, properly speaking, is never met with in this cavity, and authors who have enumerated it among the diseases that occur here, have evidently mistaken mucous engorgement for it. The fluids that accumulate here are of a mucous or muco-purulent character, except when they are the result of the disorganization of some of the surrounding parts; then they are sanious.

Previously to the discovery of this cavity, the diseases of the maxillary sinus were little understood; and though their distressing and often fatal effects must have been frequently observed, their real seat was not known, and consequently the means employed for their cure could seldom have succeeded in accomplishing it. They were thought to have originated within the substance of the osseous tissue, and I have no doubt, that many of the supposed cases of *spina ventosa* and *tumours* of the superior maxillary bone, spoken of by some of the older writers, were morbid affections of this cavity. Even subsequently to the knowledge of the existence of this antrum, similar mistakes seem to have been committed. Many cases of disease of the superior maxilla are narrated by authors without any mention, or even allusion to this cavity, which, from the description given of them, were obviously seated in it. Several such are recorded by *Wiseman*, in his treatise on surgery, published in 1676.† But notwithstanding his seeming ignorance of the seat of these affections, he evidently had a pretty correct idea of their curative indications, for the plan of treatment which he adopted differs but little from that pursued at the present day.

The most simple form of disease that occurs in this cavity, is inflammation of its lining membrane, and this in most instances may be said to precede all others. It often subsides spontaneously, but when it continues for a long time, it is apt to become chronic, and then it not unfrequently gives rise to other and more formidable kinds of disease. When unattended by any other morbid affection, either local or constitutional, it is easily cured.

* Vide, Traite des Maladies des Fosses Nazales et de leurs Sinus; art. v. sec. 2, page 226.

† Vide, Book iv. chap. iv. Obs. 12, 13 and 17, pages 265, 280 and 281.

A purulent condition of the fluids of the antrum is a very common affection, but it is seldom met with in persons of good constitutions. It seems to be dependant upon a bad habit of body and inflammation of the pituitary membrane of the sinus, which last results more frequently from dental irritation than any other cause. This condition of the secretions of the antrum, sometimes gives rise to caries and exfoliation of portions of the surrounding bone, and to fistulous ulcers, but when it is dependent upon no other local cause than simple inflammation of the mucous membrane, it is seldom that such effects result from it, but when complicated with other morbid conditions of the cavity, they are not unfrequent.

All purulent conditions of the secretions of the pituitary membrane of this cavity, are by some denominated abscess. The name however, as is justly remarked by Mr. Thos. Bell, is improper. Abscess is a different affection, and it is one, that very seldom occurs here; yet, instances of it have been met with, at the extremities of the roots of teeth that had perforated the sinus, and it sometimes happens that when an abscess is seated in the alveolus of a superior molaris, the matter, instead of making for itself a passage through the socket of the tooth on either side, escapes into this cavity, and finally with its secretions, through the nasal opening. Mr. Bell, the gentleman, whose opinion I just referred to, describes a case of abscess seated in the upper part of the antrum,* but this as he himself states, is the only one of the kind on record.

Ulceration of the lining membrane is an affection less frequently met with than either of the preceding. It is rarely, if ever idiopathic, but seems rather to be dependent, both upon some other local malady and some specific constitutional vice. Scorbutic and scrofulous dispositions and those in which there exists a venereal taint, are by far more liable to be affected with ulceration of this membrane than persons of sound constitutions, and consequently local remedies alone, are seldom adequate to its cure. It is almost always complicated with fungi of the membrane and caries of the walls of the sinus, and when neglected for a long time, it sometimes takes on a cancerous form and becomes incurable

*Anat. Phys. and diseases of the teeth, page 269.

Next in the order of arrangement, which I propose to adopt in treating upon the diseases of this cavity, is caries of its walls. This, though always complicated with one or more other forms of diseased action, seems nevertheless to be worthy of separate consideration. Like ulceration of the lining membrane, it is an effect of some one or more other affections. It may result from accumulation of the secretions of the sinus, ulceration, or from tumors.

The occurrence of fungus and other kinds of tumors is less frequent than any of the preceding affections, yet this cavity is not exempt from them, and they constitute the most dangerous description of malady that the superior maxilla is subject to. Although is it probable, that in their incipient stage, they might in nearly every instance be radically removed, it is seldom that they are cured after they have attained a very large size, and implicated in their morbid action to a considerable extent the circumjacent tissues. They have, however, been successfully extirpated even after they had acquired so great a volume as to have implicated to such an extent, the surrounding parts, as to have rendered necessary the removal of the whole of the superior maxillary bone. They usually grow with great rapidity, and when not radically removed, are generally soon reproduced.

Besides these, other varieties of disease are occasionally met with here, and the antrum is liable to injuries, from blows and other kinds of mechanical violence, and from the introduction of insects and other foreign bodies; but of these, it is not now necessary to to speak, as they will hereafter come up for special consideration.

In regard to the causes of the various morbid affections of the maxillary sinus, there exists some diversity of opinion. It is thought by some that these diseases often result from certain specific constitutional vices, independently of any local agency whatever; others attribute them to the obliteration of the opening of this cavity into the nose; some again suppose them to be dependent upon the same causes as are the diseases of the nasal fossæ, while others contend that they are produced by dental irritation. Now, that all of these may exert an influence upon this cavity, is undeniable; but that some do not do it to the extent, and in the manner which many seem to suppose, is clear; and, while I do not profess to be at all singular in the views which I entertain upon this part of the subject, I hope to be able to point out some errors, into which, not a few have evidently fallen. Opinions are frequently formed or adopted, and conclusions arrived at, that are neither warranted by facts nor supported by sound philosophy, and as it is important to a correct knowledge of the therapeutical indications of, at least, most diseases, that their causes be understood, I shall endeavour, in the course of the present treatise, to point out those which are principally concerned in the production of the several morbid affections of the maxillary sinus, or rather to explain the different kinds of influence, that those already mentioned, are capable of exerting upon this cavity, for, as I have before stated, I do not deny that it is to the presence of some one or more of these, that the diseases to which it is liable are attributable.

In carrying out this design, it is not my intention to indulge in vague speculation; I shall rely principally upon facts, to establish the views which I propose to advocate, for one fact, is worth a dozen theories based upon no better foundation than mere hypothesis; and although the detail of these be tedious and comparatively uninteresting, it is only by their aid that we can hope to arrive at truth, and it is this, that those, to whom the treatment of diseases involving life, is committed, should most desire.

The effects exerted upon this cavity by a bad habit of body or constitutional vice, are not such as many seem to suppose. They do not amount to perceptible manifestations of disease; they only consist in an increase of the susceptibility of the tissues of which it is composed, to morbid impressions, and when an unhealthy action is lit up in it, in a more aggravated, and not unfrequently different form of disease than that which would otherwise have been produced. And, not only is the susceptibility of this cavity to morbid impressions increased by an ill habit of body, or constitutional vice, but that also, not unfrequently, of every part of the whole organization; and this increase of susceptibility, may exist for years, and not result in marked demonstrations of either general or local disease. But, if the body, or any part of it, in the meantime, be subjected to the action of morbid irritants, disease, either general or local, according as the whole or only a part of it is acted upon by them, will be the result. Again, the same cause of irritation acting upon another, in whom there exists no constitutional vice, and who, as a consequence, is not possessed of so great an aptitude to be morbidly excited, might not determine any appreciable unhealthy action.

Thus it may be seen, that disease of the maxillary sinus is dependent upon some other determining cause than a particular disposition or vice of the general system; and yet, without this, no morbid effects might be produced, or if produced, they would be of a different and less aggravated character. Any disposition or vice of body, which weakens the vital energies of the system, increases the susceptibility, or what in medical language it would be more proper to term, excitability of all its parts endowed with sensibility, those of this cavity equally with the rest. There are various kinds which have this effect: as for example, the scorbutic, scrofulous, venereal, mercurial, &c. &c., each of which, may influence the character of the morbid action excited in it, in a manner peculiar to itself, or similar to that which might be exercised by another, and cause it to assume a greater or less degree of malignancy, according as the functional operations of the body generally are more or less enervated by it.

This seems to be the only way in which a bad habit of body, is capable of affecting the maxillary sinus; it is a predisposing, but not an exciting cause of disease, and it is important that this distinction should be borne in mind,—the one, should never be confounded with the other, because an error of this sort, might, and would in many instances, lead to the adoption of incorrect views concerning the therapeutical indications of the disease.

I might enlarge upon this part of the subject, but it is not necessary to do so now, inasmuch as I shall have occasion to advert to it hereafter.

Inflammation and even ulceration of the pituitary membrane of the nose, sometimes extend themselves to the maxillary sinus, but a morbid action is not so frequently excited in this cavity, by an unhealthy condition of the nose, as the intimate relationship subsisting between the two, might lead some to believe. It is seldom that both are affected at the same time, and hence I infer, that, although lined by one common membrane, the propagation of disease from one to the other, is an occurrence, which but rarely happens.

But although the diseases of the nasal fossæ, may occasionally give rise to a morbid condition of the maxillary sinus, it is questionable whether the exciting causes of the affections here met with, ever act directly upon this cavity. Concealed as it is, within the substance of the superior maxilla, and communicating with the nose only by means of a very small opening, it would seem to be beyond the reach, of at least, most of the causes, to the action of which, the diseases of the nasal fossæ are attributed.

The obliteration of the opening of this cavity, is sometimes caused by disease in the nose, and when this happens, it is followed by a mucous engorgement of the sinus, inflammation of its lining membrane, distention of its osseous walls, and not unfrequently by other and more complicated forms of disease. But the closing of this opening is oftener an effect than a cause of disease in this cavity, and it generally re-establishes itself without any assistance of art, after the cure of the affection which caused it.

If all the circumstances connected with the history of the diseases under consideration could be ascertained, I think it would be found that these affections are more frequently originated by a morbid condition of the teeth, gums and alveolar processes, than any other cause. There are no sources of irritation to which this cavity is so much and so often exposed, as that of the dental organism. It is separated from the apices of the roots of the superior molares and bicuspides only by a very thin plate of bone, and it is sometimes even penetrated by them,* so that it could

* Some are of the opinion that the maxillary sinus is never penetrated by the roots of any of the teeth, except in cases where disease has destroyed the thin plate of bone which usually covers the apices of those immediately beneath it; but that this is incorrect, is proven by the fact, that both antra are often perforated by them, which would hardly be the case were the perforations the result of disease, for it rarely happens that both cavities are affected at the same time. Indeed, I have never, except in a solitary instance, known one antrum to be perforated by the roots of the teeth, when the other was not similarly penetrated, and it is by no means uncommon for the floor of this cavity to be pierced by the apices of the roots of the first and second superior molares.

scarcely be otherwise than that aggravated and protracted disease in the teeth and alveoli which are beneath it, should exert an unhealthy influence upon it. The pain occasioned by diseased teeth, is often very severe, sometimes almost excruciating, and the inflammation excited by them in the alveolo-dental periostea and gums, frequently extends itself to the whole of one side of the face. It could hardly be possible therefore, for this cavity to escape. Alveolar abscess, and sometimes necrosis and exfoliation of the socket of the affected tooth, result from the inflammation thus lit up. It often happens too, that the gums and alveolar periosteum are affected for years with chronic inflammation, and other morbid affections, which it is not now necessary to enumerate.

If, in addition to these facts, other proofs be necessary to establish the agency of dental and alveolar irritation in the production of disease in the maxillary sinus, they may be had. Many of the affections that are here met with, are often cured by the removal of diseased teeth after other remedies have been employed in vain, and that too, without even perforating the antrum. This would not be the case, if the irritation arising from them did not extend to this cavity, and if the disease in it were not dependant upon the irritation produced by them.

Most writers on these affections agree in ascribing them to a morbid condition of the teeth and alveoli. There are some, however, who though they admit that dental irritation may perhaps occasionally give rise to them, seem nevertheless to attribute their occurrence in the majority of instances to other causes, such as irregular exposure to cold, blows upon the face, and certain constitutional diseases. Others again assert, that "if we except caries of the teeth, and the diseases of the dental periosteum, which can be communicated to the pituitary membrane, the causes of these diseases are unknown."* These are important exceptions, but that they are sometimes traceable to other causes, there can be no question. It is very certain, however, that these give rise to them more frequently than any other, and in their treatment, the state of the health of the teeth and alveoli should never be overlooked.

* Vide, Traite des Maladies des Fosses Nazales et de leurs Sinus; par I. L. Deschamps, fil, art. v. sec. 2, page 226.

I shall now proceed to the consideration of the different affections of this cavity, under their respective and appropriate heads.

### OF INFLAMMATION OF ITS LINING MEMBRANE.

As has before been intimated, inflammation when not complicated with any other morbid affection, is the most simple form of disease to which the pituitary membrane of the antrum maxillare is subject. It precedes and accompanies all others that are here met with, and it will therefore be proper to offer a few remarks upon it, before entering upon the consideration of those of a more aggravated nature.

Shielded, as the pituitary membrane of this cavity is, from most of the acrid and irritating agents to which it is exposed in the nasal fossæ and some of the other cavities of the body, it would rarely here be affected with inflammation, were it not that it is frequently acted upon by other morbid excitants, whose immediate influence seldom extends beyond the alveolar border and face. But, exposed as it is, to the action of irritants of a very aggravating kind, inflammation of it, is of frequent occurrence—more so perhaps, than many are aware of, and it is to this, that those deep seated pains in the superior maxilla and face, which are usually denominated rheumatic, are, in many instances, attributable.

Inflammation of the lining membrane of the maxillary sinus, when not complicated with any general morbid tendency, or constitutional predisposition, seldom gives rise to any other form of diseased action; and it usually subsides spontaneously on the removal of the cause that induced it. This membrane in good constitutions, is less subject to inflammation, and as a consequence, to any other description of morbid action, than those in whom there exists some vice of body, or unhealthy predisposition. Febrile and gastric affections, eruptive diseases, such as measles, small-pox, &c. &c. syphilis, an excessive and protracted use of mercurial medicines, a scorbutic or scrofulous diathesis of the general system, and in short, every thing that has a tendency to enervate the vital powers of the body, increases its irritability.

When in a healthy condition, it secretes a slightly glutinous, transparent and inodorous fluid, by which it is constantly lubricated, but inflammation in it, changes its secretions; it causes them to become vitiated; at first, to be less abundant, afterwards to be secreted in larger quantities than usual, to be more serous, and so acrid as sometimes to irritate the membrane of the nose over which they pass after having escaped from the antrum. It also causes them to exhale an odour more or less offensive, according as the inflammation is severe or mild. It moreover gives rise to an engorgement and thickening of the membrane, and sometimes to an obliteration of the nasal opening. This last, however, rarely occurs, but when it does happen, an accumulation of the secretions and other morbid phenomena, of which I shall hereafter treat, result from it, as a necessary consequence.

Inflammation of the pituitary membrane of this cavity, is sometimes followed by inflammation of that of the nasal fossæ; but I am of the opinion, that when this happens, it is occasioned by the acrid qualities of the fluids that are discharged from the sinus into the nose.

If at any time during the continuance of the inflammation, the patient is attacked with severe constitutional disease, the local affection will be aggravated and sometimes caused to assume a different character.

The inflammation when long continued, degenerates into a chronic form, and is sometimes kept up for several years, without giving rise to any other unpleasant effects, than occasional paroxysms of a dull and seemingly deep-seated pain in the face, and a vitiated condition of the fluids that are secreted in this cavity. The slightly fetid odour which they exhale, ceases to be annoying or even perceptible to the patient, as he becomes accustomed to it.

*Symptoms.*—The symptoms by which this affection is characterized, though not always precisely the same, are nevertheless, for the most part, very similar. Boyer describes them to be severe, fixed, and deep-seated pain under the cheek, extending from the alveolar border to the lower part of the orbit, local heat, pulsation, and sometimes fever.* He, however, tells us that these symptoms are not always present, and that the disease sometimes exists when it is not suspected. I apprehend that this is never the case.

* Vide, Traite des Maladies Chirurgicales, &c. &c. tom. vi. art. 3, page 138.

Other affections of the face and superior maxillary, may be mistaken for this, and this for others, but that it should exist without being attended with pain or any other signs indicative of its presence, I cannot believe to be true, and I cannot imagine how he arrived at such a conclusion.

Deschamps distinguishes the symptoms from those of other affections of this cavity by "a dull, heavy pain in the region of it," which he says, "becomes sharp and lancinating," and extends from the alveolar arch to the frontal sinus. The disease goes on without interruption, increasing until the superior maxilla of the affected side is more or less involved. This malady he tells us cannot be confounded with any other, if there is no external visible cause; it differs, he says, from a retention of mucus, by being painful at the commencement, and by not being accompanied with swelling of the bones;" he distinguishes it from polypus, as that causes no pain, and from cancer, which occasions pain of a different kind. "Suppuration and ulcers have peculiar signs which cannot be confounded with those of inflammation." Pain in the molar, and bicuspid teeth, accompanied by a sense of fluctuation in the parts, he seems to regard as a very certain indication of inflammation, and especially when joined to the other symptoms. "If an external cause, is discovered, it" he says, "will furnish a certain diagnosis,"* he also mentions fever and headache as almost invariable accompaniments.

The inflammation, if not subdued by appropriate remedies, after having continued for a length of time, gradually assumes a chronic form; the pain then begins to diminish, and is less constant; it becomes more dull, and is principally confined to the region of the antrum. The teeth of the affected side cease to ache, or ache only at times, they still, however, remain sensitive to the touch. The mucous membrane of the nostril next the diseased sinus, is often tender and slightly inflamed, and if the other one be closed in the morning, or after two or three hours sleep, by pressing upon it with the thumb or one of the fingers, and a violent expiration made through this, a thin watery fluid, of a slightly fetid odour, will be discharged, and pain will be experienced in the region of the antrum.

*Traite des Maladies des Fosses Nazales et de leurs Sinus; sec. 2, art. ii. pages 236–7.

*Causes.*—The explanation of the causes of inflammation of the pituitary membrane of the maxillary sinus, as given by Deschamps, is far from satisfactory. After stating that it is produced by all those general causes which give rise to it in other parts of the body," he enumerates among, what he denominates, "peculiar causes," a degeneration of the humour which it pours out, "blows upon the cheek, fractures, wounds, and the extraction of teeth."* What he means by those general causes, I am at a loss to understand, except it be certain morbid dispositions of body, irregular exposures to cold, &c. &c. The influence which these exert upon the mucous membrane of this cavity, has been explained in another place: it will therefore be unnecessary to repeat what has already been said concerning them, except it be to state that they first exercise the same influence upon the membrane in other parts of the body, that they do here, and that this consists, not in actual disease, but only in an increase of excitability. The exciting cause of inflammation of this cavity, is local irritation, and, a degenerated condition of its secretions, blows upon the cheek, wounds and the extraction of teeth, may be enumerated among the agencies which are sometimes concerned in its production.

Boyer says, "This inflammation may be produced by a blow upon the cheek, by small-pox, measles," &c. but the most ordinary cause is caries and pain in the teeth.† The two last, although not mentioned by Deschamps, are very frequently concerned in the production of this affection. All morbid conditions of the teeth, in fact, and of the gums, that give rise to irritation in the alveolar periosteal tissue, may be regarded as among the most frequent of its exciting causes. And, of the affections of the teeth that do this, caries, necrosis and exostosis may be mentioned; also, loose teeth, and the roots of teeth that have either been fractured in an attempt at extraction, or by a blow or fall, and left in their sockets, or that have remained after the destruction of their crowns by decay. It sometimes happens too, that inflammation is excited in this membrane by fractured alveoli, but when an accident of this sort occurs, the detached portions of bone are generally soon

* Traite des Maladies des Fosses Nazales et de leurs Sinus; Art. 2, page 236.

† Traite des Maladies Chirurgicales, &c. &c. Art. iii. tom. 6, p. 138.

thrown off by the operations of the economy, and the cause being removed, the inflammation immediately subsides. But not so with the roots of teeth. They often remain concealed in their sockets for years, except they be removed by art. Nature, it is true, makes an effort to expel them from the jaw, but this is accomplished only by a slow and very tedious process, and not, in many instances, until they have given rise to some serious affection. But, of the deleterious effects that result from roots of teeth in the alveoli, it is not necessary now to speak; as extraneous bodies they are always productive of more or less irritation.

As to the influence which the diseases of the gums exert upon the alveolar periosteum, suffice it to say, that it is little, if any less hurtful than that resulting from the affections of the teeth. Disease here is very certain to communicate itself to that membrane. It is, in fact, to the morbid affections of this structure, that the diseases of the alveoli, are most frequently attributable. It is true, the diseases of the gums, do not often give rise to acute alveolar periostitis, but they excite in the lining membrane of these cavities, a chronic inflammation, which, if not arrested, sooner or later eventuates in their destruction, and as a consequence, to the gradual loosening, and ultimately, loss of the tooth.

Thus it may be perceived, how very liable the periosteal tissue of the alveolar cavities is to become inflamed; and, inflammation having been lit up here, how very easy it would be for it to extend itself to the maxillary sinus; and that it often does propagate itself to this cavity, no one, I should suppose, could doubt. But, we are not left to mere conjecture, or doubtful inference, in regard to this matter. Not only is inflammation, but every other form of disease to which this cavity is subject, more frequently traceable to alveolo-dental irritation, than to any other cause. The truth of this assertion is established by the result of the treatment of cases which I shall hereafter have occasion to notice.

Having said thus much concerning the causes of inflammation of the lining membrane of the antrum maxillare, I shall now proceed to describe its remedial indications.

*Treatment.*—The curative indications of the affection under consideration are simple, and for the most part, similar to those of inflammation in other parts of the body. "Bleeding from the arm, feet, pediluvia, antiphlogistics, mild purgatives, emollient cataplasms, anodyne applications to the cheek, fumigations to the nose, by means of an inverted funnel," says *Deschamps,** are the means usually employed. Originating, however, as does most frequently inflammation of the lining membrane of the maxillary sinus, from the irritation produced by decayed, dead or loose teeth, the removal of these, will, in most cases, be all that is required to accomplish a cure. This is the practice which Boyer recommends,† and Deschamps says, "it is not uncommon for the disease to cease immediately after the extraction of an affected tooth."‡ But when the inflammation is severe, its reduction will be expedited by bleeding from the arm, saline purgatives and fomentations to the face. In many cases, great benefit will be derived from the application of leeches to the cheek, as recommended by Mr. Thos. Bell.§ I have known the most decided advantage to result from their employment; but, when the disease is dependent, as it in most instances is, upon an unhealthy condition of the alveolar process, the first thing to be done, is to remove from them all such teeth or roots of teeth, as are productive of the least irritation, for, while any local sources of irritation are permitted to remain, neither topical nor general bleeding, nor any other treatment will be of permanent advantage.

The treatment of inflammation of this cavity, however, may be best illustrated by the following cases, which, from very many similar ones, that have fallen under the author's notice, he will briefly narrate.

Case I.—In the spring of 1840, W. H., æt. nineteen, of a sanguino-scorbutic temperament, called to consult me concerning a deep-seated pain, which he had felt, for more than two months, in his left cheek. On interrogating him, I ascertained that he had occasionally, during this time, discharged a fetid matter from the nostril of the affected side. For the relief of the pain in his cheek, leeches and anodyne fomentations to the face, and purgatives had been prescribed, but from these he obtained only

* Vide, Maladies des Fosses Nazales, sec. 2, Art. ii. p. 238.

† Vide Maladies Chirurgicale, tom. vi. page 139.

‡ Maladies des Fosses Nazales, p. 238.

§ Anatomy Phys. and diseases of the Teeth, p. 251.

temporary relief. At length, his medical attendant, suspecting that the affection was in some way connected with his teeth, which were in a very unhealthy condition, advised him to obtain my opinion with regard to the agency that they might have in its production.

The first and second molares, the third not having yet appeared, and the two bicuspides, on examination, I found so much decayed, as to render their restoration to health impossible. Abscesses had formed in the sockets of the first bicuspis and second molaris, and the matter from which was almost constantly discharging itself from fistulous openings through the gum beneath the upper lip and cheek. Both the bicuspides and molaris of this side were sensitive to the touch, and the gum around them was in a spongy and inflamed condition.

Not being able to institute any treatment that would be of service to his teeth, and convinced that the affection of his cheek, was attributable to the irritation which they produced, I advised their immediate extraction, to which operation he at once submitted. Four weeks afterwards, he called and informed me that the pain in his cheek subsided about ten days after the removal of his teeth, and that it had not since returned.

Case II.—Mrs. L——, æt. about twenty-seven or eight, of a scrofulous habit, had been at times affected, for more than two years, with a deep-seated pain in the right side of her face, midway between the orbit and alveolar ridge; and on closing the left nostril, and making a violent expiration through the right, discharged a slightly, yet perceptibly fetid mucus matter, which occasionally excoriated the mucous membrane lining this cavity of the nose. This pain, from the fact that it was most severe in cold and damp weather, was thought to be rheumatic. General and local bleeding, fomentations, mustard plasters, purgatives, anodynes, tonics, and many other remedies were in vain employed.

A severe paroxysm of tooth-ache about this time, July, 1841, more than two years subsequently to the time when she first felt the deep-seated pain in her cheek, induced her to apply to me for my professional services. On examining her mouth, the crowns of the second molaris, dens sapientia, and first bicuspis of the affected side were destroyed by caries; the gums covering the sockets of their roots, were inflamed and very sensitive. It was the roots of the wisdom or third molar tooth that ached, and for the alleviation of the pain of which, she had called upon me. Extraction being the only remedy that held out the least prospect of relief, I at once proposed that operation, and at the same time urged upon her, the importance of having the roots of the second molaris and first bicuspis removed. A great deal of persuasion however, was necessary to obtain her consent, she being of an exceedingly nervous and timid disposition, but having made up her mind to submit to the operation, she determined to have it immediately performed. She had no cause to regret their removal, for not only was she freed from the annoyance which they occasioned to her tongue, gums, &c. but the operation was likewise followed by a speedy subsidence of the pain in the cheek, and a cessation of the fetid discharge from her nose.

CASE III.—In December, 1841, I was consulted, by Mr. S. M. J——, twenty-three years of age, and of sanguinous disposition. He had been affected for several months, with a dull heavy pain, which as he said, seemed to be seated deep in his right cheek, and as in the case last described, a fetid mucus matter was discharged from the nostril of the affected side, on making a violent expiration through it, with the other nasal cavity closed. His teeth, to all appearance were perfectly sound, but the gums around the first and second bicuspides and first molaris, were inflamed, spongy, and slightly ulcerated between their edges and the necks of the teeth, from which, they had separated up to the edges of the alveoli. These morbid phenomena, he attributed to a blow, which he had received from a fall, upon these teeth about two years before. It was immediately followed by pain, inflammation, and in about two months, by the exfoliation of several small portions of the alveolar processes, which came out through the gum. These were the only unpleasant effects which he experienced at the time, but there was always afterwards, a slight soreness in the teeth, which had received the injury. This, as he informed me, gradually extended itself higher and higher up into the substance of the jaw, until, about four months previously to his having called on me, when its place, seemed to be taken, by the kind of pain first described; and soon after the fetid discharge from the right nostril was discovered.

That the deep-seated pain in the right superior maxillary, was occasioned by inflammation of the mucous membrane which lined its sinus, I could not doubt, and that this had resulted from the alveolar irritation, caused by the violence that had been inflicted upon the first and second bicuspides and first molares, to me, was equally evident, and under such circumstances, the removal of the injured teeth, seemed to constitute the proper curative indications, I therefore, at once, proposed their extraction, which operation he immediately submitted to. Three weeks after, the pain in his jaw had entirely disappeared.

I could mention a number of similar cases, where the pain and other inflammatory symptoms, have been relieved simply by the removal of decayed and dead teeth. When the inflammation is dependent upon the presence of these, and that it is in a large majority of cases, there can be no question, their extraction will suffice in nearly every instance, to effect a cure; but when it has been produced by any other cause, as for example, a sudden exposure to cold, a blow upon the cheek, fractures of the alveoli, &c. &c. then the application of leeches to the gums and cheek, fomentations, bleeding from the arm, purgatives and other treatment may be called for.

This affection, as is remarked by Boyer,* would be of little consequence, were it not that it is liable to give rise to other and more dangerous forms of disease, such, for instance, as a purulent condition of the secretions of this cavity or an engorgement of it. It should never therefore, be permitted to continue, but should be as speedily arrested as possible; and for the accomplishment of this, the means here pointed out, will, if timely and properly applied, be found fully adequate.

Inflammation of the pituitary membrane of the maxillary antrum, sometimes causes the opening into the nose to become closed, and when this happens, an engorgement of the cavity is certain to result. Various other morbid phenomena also occasionally arise from it, but the most common form of disease, resulting from it, is an altered or purulent condition of the mucous secretions of the cavity, and it is this, which I propose next to notice.

* Maladies Chirurgicale, tom. vi. page 139.

### OF A PURULENT CONDITION OF ITS SECRETIONS AND ENGORGEMENT.

A purulent condition of the secretions of the maxillary sinus and mucous engorgement, are indiscriminately, though very improperly, denominated by most writers on the affections of this cavity, abscess. To this, neither bears the slightest resemblance. Deschamps, treats of the former under the name of suppuration, and the latter under that of dropsy.* In speaking of the first he tells us, "if after the time of the inflammation has passed, the surrounding parts cease to be painful, while the affection still continues to cause pain in the antrum, and the fever, though diminished, occurs at irregular intervals, and if the inflammation is followed by a pulsating pain, we will have reason to suppose that an abscess has formed in the sinus; and," he continues, "all doubts will be removed, if on the patient's inclining his head to the opposite side, matter is discharged into the nostril, or if some tubercles are formed near the outer angle of the eye, or alveolar border, which last happens more frequently; and finally, if the purulent matter not finding any opening through which to evacuate itself, distends the sinus to such an extent as to form a tumour outwardly upon the cheek." In short, all the symptoms which he mentions as belonging to the disease, are those that accompany the one now under consideration. The matter he says is of a "putrid serous consistency."

Bordenave has fallen into a similar error. He too, terms an altered state of these secretions, suppuration of the membrane, and he tells us that inflammation is not necessary to it. He seems moreover, to have confounded in alveolar abscess those cases where the matter, instead of evacuating itself as it ordinarily does, through an opening which it makes for its exit through the alveolus and gum into the mouth, escapes into the antrum, with abscess of that cavity. Again, he asserts that the disease, (suppuration as he calls it) may be independent of the surrounding parts, and although ordinarily implicated with an altered

* Vide Maladies des Fosses Nazales, pages 227 and 239.

condition of them, he affirms, it is sometimes the effect of disease primarily seated in this cavity.*

There is no doubt that a purulent condition of the fluids of this cavity is often complicated with ulceration of the lining membrane, but that the affection is at all analagous to abscess or suppuration, its very nature and situation, is sufficient to disprove. "A reference to the structure of the antrum," says Mr. Bell, "would appear to be sufficient to point out the improbability, to say the least, of the occurrence of abscess in such a situation. That a mucous membrane covering, in a thin layer, the whole internal surface of such a cavity, should become the seat of all the consecutive steps of true abscess, is a statement bearing on the face of it an obvious absurdity."† But notwithstanding the seeming improbability of such an occurrence, and it is certainly one that very rarely happens, abscess does nevertheless sometimes seat itself in this cavity; but, it is a different affection altogether from that usually treated of under that name. I have already adverted to a case narrated by Mr. B., a description of which, I intend hereafter to give.

When complicated with ulceration of the mucous membrane, and it is probable that a purulent condition of the secretions of this cavity, in most instances is thus complicated, the affection is precisely analagous to ozena, and, by many of the older writers, it is designated by that name. Mr. Bell describes it, and very properly too, as being similar to gonorrhœa—both diseases equally consisting of an altered secretion, in the one, of the pituitary membrane, and in the other of the mucous lining of the urethra, which, in neither instance possesses any of the characteristics of abscess, though the matter in both is purulent.‡

It has been before stated that the obliteration of the nasal opening was more frequently an effect than a cause of disease in the maxillary sinus; it does, however, sometimes become closed, from other causes than an unhealthy condition of this cavity, and

* Vide Memoirs de l' Acadcmie Royale de Chirurgico, vol. 12, p. 8. ed. 12mo.

‡ Anat. Phys. and Diseases of the Teeth, page 253.

† Bell on the Teeth, page 254.

when this happens, an engorgement of the sinus is the inevitable consequence, but the fluids thus accumulated are not always at first purulent. They however soon become so, by their retention in the cavity, and, when the closing of the opening is the result of previous disease in the antrum, the secretions are more or less altered from the very first.

The accumulation of the secretions of the antrum, whether in a healthy or purulent state, is a constant source of irritation to the lining membrane, and the pressure which they ultimately exert upon the surrounding walls, causes a new form of diseased action to be set up, that not unfrequently involves the whole of the bones of the face as well as those of the base of the cranium, and which, if not soon arrested, ultimately destroys the life of the patient. They, however, when prevented from escaping through the nasal opening, eventually make one through which to evacuate themselves, and this is sometimes effected through the cheek, at other times beneath it, just above the alveolar ridge, or through the palatine arch or alveoli down by the sides of the roots of one or more of the teeth, and thus establish a fistula, from which a fetid matter will be almost constantly discharging itself. From openings of this sort, the matter is sometimes evacuated for years, while the disease in the antrum, in the meantime, very frequently, does not seem to undergo any apparent change. At other times the membrane ulcerates and the bony walls become carious, and cause a fetid sanies to be almost continually discharged.

But, a purulent condition of the mucous fluids of this cavity, independently of caries of the bone, or even of simple fistulous openings, is an exceedingly troublesome and unpleasant affection. The odour from the matter is of itself, often very annoying even to the patient, and when the secretions are retained for some days in the sinus before they are evacuated, the fetor from them is sometimes almost insufferable.

In good constitutions, it is rare that the secretions, of the antrum, become purulent, even though they be confined for a long time in the cavity. It is only in scrofulous, scorbutic, or debilitated habits that they are liable to become thus altered. Inflammation of the lining membrane, the immediate or proximate cause, may exist for years without giving rise to it. The differences in the effects produced upon them, and the surrounding parts, by inflammation of this membrane is owing to the differences in the state of the constitutional health of those affected by it.

Where a puriform state of the secretions of this cavity is complicated with ulceration of the membrane, the matter will have mixed with it a greater or less quantity of flocculi, sometimes of so firm a consistence, as to block up the nasal opening, and prevent its exit. Mr. T. Bell says, he has seen more than one case in which a considerable accumulation had taken place in the antrum, accompanied by the usual indications of this affection, (muco-purulent engorgement of the sinus) when a sudden discharge of the contents into the nose, took place, "in consequence of the pressure having overcome the resistance which had thus been offered to its escape."* Cases of a very similar nature have fallen under my own observation, the history of some of which may be given in the course of this essay. The formation of these flocculi rarely cease, except with the cure of the ulcers of the membrane; they give rise to considerable irritation, and their presence always constitutes an obstacle to the cure. They are, however, usually easily removed by injections.

The pituitary membrane of the antrum when in a healthy state secretes, as I have before stated, a transparent, slightly glutinous and inodorous fluid, which is poured out only in just sufficient quantities to lubricate the cavity. But, no sooner is inflammation excited in the membrane, than its secretions become more abundant, and, at first thinner, but afterwards thicker and more glutinous,† but their colour and consistence are not always the same. Instead of being transparent, they sometimes have a dirty opaque appearance; at other times they assume a greenish, whitish or yellowish colour, and in some instances they bear a considerable resemblance to pus, which, it has been conjectured, might be owing to a suppuration of some of the mucous follicles of the lining membrane of the antrum, and a consequent mixture of pus with its secretions. Mr. Bell, however, inclines to the opinion that it

* Vide, Anat. Phys. and Diseases of the Teeth, p. 258.

† Vide, Maladies Chirurgicale, tom. vi. p. 140.

is attributable to an "alteration simply" of the secretions of the cavity.* But their colour and consistence, I am disposed to believe, are determined by the degree of inflammation, the length of time it has existed, the state of the health of the lining membrane, and that of the surrounding osseous walls, the egress which the matter has from the sinus, and the general habit of the body.

Mucous engorgement of the maxillary sinus and purulent accumulations, it has been remarked, are more common to young subjects than to middle aged ones, or persons in advanced life. An eminent French writer says, that of three individuals affected with dropsy (mucous engorgement) the oldest was not twenty years of age.† Although these affections are more common to young persons than individuals of advanced life, they are by no means confined to the former. Debilitated habits, though not equally, of every age, are subject to them.

*Symptoms.*—The diagnostics of the several affections of the antrum, as has been intimated in a preceding place, are so much alike, that it is often difficult to distinguish those that belong to one from those that are attendant upon another. The symptoms of mucous engorgement and purulent accumulations, however, are generally such, as will enable the practitioner to distinguish, with considerable certainty, these affections from any others that are here met with. They are always preceded by inflammation of the lining membrane; a description of the signs of which, having already been given, I need not repeat. Omitting these then, I will at once proceed to mention those by which they are accompanied.

In speaking of those which more particularly belong to a purulent condition of the secretions of the antrum. Deschamps says, the affection may be distinguished by a dull heavy pain, extending along the alveolar border; but, upon this symptom alone, little reliance can be placed; as it is always present in chronic inflammation of the pituitary membrane of this cavity. But in addition to this, he mentions the presence of decayed teeth, soreness in those that are sound, and on the patient's inclining his head to the side opposite the one affected, the discharge of fetid matter

* Vide, Anat. Phys. and Diseases of the Teeth, p. 254.

† Vide, Traite des Maladies Chirurgicales et des Operations qui leur conviennent, tom. vi. p. 139.

from the nose.* These are certainly very conclusive indications of purulent effusion in this cavity. Bordenave, after enumerating the symptoms indicative of inflammation, mentions the following as belonging to the affection of which I am now speaking; viz. dull and constant pain in the sinus, extending from the maxillary fossæ to the orbit; a discharge of fetid matter from the nose, when the patient inclines his head to the opposite side, or when the nose is blown from the nostril of the affected side.† These are the symptoms which are mentioned by almost every writer upon the subject, as indicative of a purulent condition of the secretions of the maxillary sinus.

The diagnostics indicative of engorgement, differ materially from those which denote simply a purulent condition of the mucous secretions of this cavity. The pain instead of being dull and heavy, as just described, becomes acute and a distressing sense of fullness and weight is felt in the cheek, accompanied by redness and tumefaction of the integuments covering the antrum.‡ The nasal opening having become closed, the fluids of the cavity gradually accumulate until they fill it, when, finding no egress, they press upon and distend the surrounding osseous walls, causing those parts which are the thinnest ultimately to give way. The effects are generally first observable anteriorly beneath the malar eminence, where a smooth hard tumour presents itself, covered by the mucous membrane of the mouth. But this is not always the point which first gives way, the sinus sometimes bursts into the orbit, at other times outwardly through the cheek, or through the palatine arch. The long continued pressure that is thus exerted upon the bony walls of this cavity, often cause them to become softened, and the calcareous molicules of those parts which first yield, to be entirely broken down.

The tumour which is at first hard, afterwards becomes soft so as readily to yield to pressure. A distention of the maxillary sinus, Deschamps says, may be distinguished from other diseases that affect the skin or intermediate structure between it and the

* Vide, Maladies des Fosses Nazales, sec. 2, art. 3, p. 240.

† Vide, Memoirs de l'Academie Royale de Chirurgie, 12mo. tom. 12, p. 10.

‡ Vide Bell on the Teeth, p. 256, see also Maladies des Fosses Nazales, page 228.

bone, by the uniformity or regularity of the tumour, its firmness at the commencement, the slowness with which it progresses, and above all, by the natural appearance of the skin, and the absence of pain when pressure is made upon the tumour. An obliteration of the nasal opening he says, may be suspected by the dryness of the nostril of the affected side.* A thickening of the mucous membrane and contraction of the nostril of the affected side, inflammation and sponginess of the gums, loosening, and sometimes, in consequence of the destruction of their sockets, displacement of the teeth, may also be mentioned as occasional accompaniments of engorgement of this cavity.†

*Causes.*—On the causes of these, in common with the other affections of the maxillary sinus, I have before spoken; it will not therefore, be necessary to say much in this place concerning them. It may be well, however, to repeat, that the secretions of this cavity rarely become purulent in individuals possessed of good constitutional health, so that it would seem, that although local irritation be necessary to it, this is capable of producing it only in those labouring under a bad habit of body, or in whom their exists a tendency to such alteration. Inflammation of the lining membrane, no matter how produced, is the immediate cause, and this, as has been before shown, results more frequently from alveolo-dental irritation, than from any other cause; and I am not the only one that is of this opinion;—it is maintained by almost every writer upon the morbid affections of this cavity. The teeth that are most frequently concerned in the production of irritation in the lining membrane of the antrum, are the first and second molares, but the bicuspides and dens sapientiæ, do sometimes cause it. But, it being conceded by nearly every one that the exciting cause of not only this, but all the diseases of this cavity is dental irritation, I need not spend time in recapitulating what has before been said upon the subject, or in controverting absurd and erroneous opinions.

Engorgement of the sinus is attributed to several causes, among which, are blows upon the cheek, caries of the teeth, &c. &c. But, whatever may be the exciting or primary causes of

* Vide Maladies des Fossess Nazales, pages 228–9.

† Vide, Anat. Phys. and Diseases of the Teeth, p. 257.

this affection, it is certain that the proximate or immediate cause, is the closing or obliteration of the nasal opening. This, like purulent secretion, may be produced by inflammation and thickening of the lining membrane of the sinus, which is perhaps the most frequent cause.*

*Treatment.*—The curative indications of muco-purulent secretion and engorgement of the maxillary sinus are, 1st, If the nasal opening be closed, the evacuation of the retained matter; 2dly, The removal of all local and exciting causes of irritation; 3dly, and lastly, the restoration of the lining membrane.†

For the fulfillment of the first, an opening must be made into the antrum, and this should be effected in that part which will afford the most easy exit to the retained matter; but as it regards the several methods that have been proposed for the accomplishment of this object, practitioners differ; and, before I proceed further, it may not be amiss to notice some of the various plans that have been adopted.

*Dr. Drake*, an English anatomist, and author of a work entitled "*Anthropologia Nova*," has the credit of being the first to propose a plan for the evacuation of accumulated fluids from this cavity, and the method adopted by him for effecting this object, consists in the extraction of a molar tooth and the perforation of the sinus through the alveolus of one of its roots. This method of treatment however, is said by some to have been inserted into Drake's anatomy by *Dr. Cowper*, an eminent anatomist and surgeon,‡ but having never seen any evidence touching the correctness of this conjecture, its truth is probably somewhat ques-

* Fauchard says, in the Anatomical Mus. of the University of Copenhagen, he saw caries of the bones of the face produced by a molar tooth, the crown of which having turned outwards, had penetrated the maxillary sinus, *Mem. de l'Academie de Chirurg*. vol. v. mem. 257. Also the fangs of the bicuspides and front molaris sometimes penetrate the sinus. Bertin Osteologie, vol. xi. p. 309.—Portal Camp. d'Anatomie Medicale, vol. i. p. 210. Note 2.—My Uerzichniss, No. 3278; there are in the Bresl. Mus. No. 8128, two teeth, as it were absorbed, which had been drawn out of the maxillary sinus.—*Otto's Compend. of Comp. Anat.*

† Vide Anat. Phys. and Diseases of the Teeth, p. 259.

‡ Heister's Surgery, note to chapter 72, p. 445.

tionable. M. Gûnz says the credit belongs to John Henry Meibomius, who a long time before proposed a very similar method of treating these affections.* Henry Meibomius, many years after the death of his father John Henry, proposed for the evacuation of accumulated fluids in the antrum, the extraction of one or several teeth.† But the perforation of the maxillary sinus through the alveolus of a molar tooth, is said not to be the most ancient method. Molinetti, as early as the year 1675, describes an opening made through the cheek into the antrum, the wall of which, after having been exposed by a crucial incision through the integuments covering it, was penetrated with a trephine. And, the perforation of this cavity through the alveolus of a superior molaris, is an operation which, according to Velpeau, was performed by Zwinger a long time before it was performed by Meibomius; and Vanuessen, says, Ruysch, extracted several molares and cauterized their sockets, for the destruction of a polypus, until an opening was made into the antrum large enough to admit the finger.‡ But Drake, according to Bordenave, seems, nevertheless, to be entitled to the credit of having been the first to perforate the maxillary sinus through the alveolus of a molar tooth, by means of a punch, for the evacuation of accumulated fluids, and the injection of the cavity. We are also informed by the same author, that Cowper treated a case of maxillary ozena, which had caused a large quantity of ichorous and fetid matter to be discharged through the nose, by extracting the first molaris and perforating the antrum through the alveolus with an instrument made for that purpose.§

It is not at all probable that Meibomius was the first to propose the perforation of the antrum through the alveolus of a molar tooth, for his researches were not published until 1718, twenty-one years after the publication of Drake's system of Anatomy, and besides, he regarded the perforation of this cavity as a dangerous operation, and on that account, confined himself simply

* Vide, Mem. de l'Acad. Royale de Chirurg. 12mo. vol. xii. p. 12.

† Vide, Discurs. de Abscessibus internis, Dresd. 1718, p. 114, and la Dissertation d'Gunz.

‡ New Elements of Operative Surgery, p. 446.

§ Mem. de l'Acad. Royale de Chirurg. vol. 12, 12mo. p. 13.

to the extraction of a tooth. Saint Yves, says Velpeau, treated with success, a person affected with fistula, the floor of whose orbit had been destroyed, by the removal of a tooth.*

With regard to the tooth most proper to be extracted, authors differ. Cheseldon preferred the first or second molaris, Junker recommends the extraction of the first or second bicuspides, and if a fistula had formed, to enlarge it instead of perforating the floor of the antrum. It is at present pretty generally conceded that the second molaris, it being directly beneath the most dependent part of the cavity, is the most suitable tooth to be removed; but, if this be sound, the first molaris, dens sapientiæ, or either of the bicuspides, if carious, should be extracted in its stead, and in fact, none of the teeth that are in an unhealthy condition should be permitted to remain.

An opening having been effected through the alveolus of a tooth into the antrum, it should be kept open until the health of the cavity be restored. For this purpose, sounds and bougies adapted to the purpose have been introduced. Heuerman, recommends the employment of a small canula, which is also preferred by Bordenave and Richter, the latter of whom says, it should be kept closed to prevent particles of food from getting into the sinus. But, whether a canula or bougie be introduced into the opening, it should be so secured as to prevent it from coming out or getting up into the antrum. Deschamps recommends that it be fastened to one of the adjoining teeth by means of a silk or metallic ligature.†

Lamorier, an eminent surgeon of Montpellier, recommended perforating the antrum immediately above the first molaris, or rather between it and the malar bone. In this, he seems to have been influenced by the consideration that the wall of the cavity here presented the least thickness, and that this was the most dependent part of the sinus. This was the point which he preferred for an opening into this cavity, but when a fistulous one had been previously formed, he did not always find it necessary to make it. The jaws being closed, the commissure of the lips are drawn outwards and slightly upwards with a curved instrument, called a speculum; this done, the gum is incised

* New Elements of Operative Surgery, p. 446.

† Vide, Maladies des Fosses Nazales, sec, 2, Art. 1, p. 234.

across the malar apophasis,* or maxillo-labial sucus,† and the bone made bare, which is next pierced with a spear-pointed punch. The opening is afterwards enlarged if found necessary.

Desault is of the opinion that the opening should be made through the canine fossa, beneath the upper lip, and for that purpose, after having laid bare the bone, he employed a sharp triangular and a blunt-pointed perforator, which he invented for the operation. Runge says, Velpeau used nothing but a scalpel. Charles Bell invented a trephine for the purpose, but this, it is thought does not possess any advantage over the instruments employed by Desault and Runge. In cases of fistula in the cheek from the antrum, Ruffel advises the insertion of a trocar, to be carried through the gum, so as to form a counter opening. Through this, in a case which he treated, he passed a seaton, and it remained six weeks; at the expiration of this time, a cure was accomplished. This practice has been followed by Callisen, Zang, Busch, Henkle, Bertrandi, Faubert and others.‡ Callisen is of the opinion that when the tumour points in the palatine arch and a fluctuation is felt, the artificial opening should be formed there. Gooch, says Velpeau, in a case which he treated, advised the perforation of the antrum through the nasal surface, and fixing in the opening a canula of lead. We are also informed by the same author, that, Acrel, after having operated in the manner proposed by Cowper, inserted a second canula into the sinus through a fistulous opening that had formed in the nose. The method attributed to Wienhold, consists in penetrating the sinus from the upper and external part of the canine fossa, with the instrument directed obliquely downwards and outwards, so as to avoid the branches of the infra-orbital nerve; and then placing in the opening thus made a little lint. Weinhold, directs, that when the antrum has no other opening, the instrument to be carried entirely through the palatine arch, and then by means of a curved needle and thread, he introduces a roll of lint, saturated or covered with some appropriate medicine, and this he designs to act as a seton.§

* Vide, Mem. de l'Acad. Royale de Chirurg. 12mo. vol. 12, p. 37.

† Vide, Velpeau's Surgery, p. 447.

‡ Vide, Mem. de l'Acad. Royale de Chirurg. 12mo. vol. 12, Obs. 18 and 21; also New Elements of Operative Surgery, p. 447.

§ New Elements of Operative Surgery, p. 448.

Velpeau says, the perforation is effected "in the point of election or of necessity. The first varies according to the ideas of the operator. The circumstances, on the contrary, determine the second. In cases of abscess, dropsy, fistulæ, and ulceration, the operation is almost always performed in the place of election. Then, provided one of the molar teeth be unsound, it must be extracted, together with the adjoining tooth; the gum is then to be cut down to the bone, externally; internally, behind and before, forming a kind of a square flap, and to be completely detached from the surrounding tissues; after this the alveoli are to be perforated with the instruments of Desault, and an opening made large enough to admit the finger into the sinus."* For the evacuation simply of purulent mucus, or accumulated fluids, I believe with Boyer,† that the opening should always be made from beneath; and, I am the more convinced of the importance of giving the alveolus of an extracted tooth the preference, from the consideration that it is to the irritation produced by some one or more of these organs, that these affections are attributable. Even though a fistula may have been formed above the alveolar ridge, beneath the cheek, or in the palatine arch, we should not neglect to extract such teeth, whether carious or sound, as may be productive of irritation. It may not always in such cases be necessary to perforate the sinus from the socket of a tooth, though the cure in most instances would be expedited by it.

Jourdain, an eminent French dentist, and graduate in surgery, instead of seeking egress for matter accumulated in the maxillary sinus, by any of these methods, proposed, in a memoir which he presented to the academy, in 1765, to probe the cavity by its natural opening and then by suitable injections to restore it to health. The academy gave this proposition its attention; it was carefully and minutely discussed. The practicability of obtaining entrance into the sinus in this way was called in question; it was contended that the difficulties presented by the peculiar structure of the parts were such that they could seldom be overcome; but to remove all doubt upon the subject, a trial was determined on.

While this subject was before the academy, M. Allouel, jr. claimed the credit of the discovery for his father, who, he said

† New Elements of Operative Surgery, p. 448.
* Vide, Maladies Chirurgicale, tom. vi. p. 149.

made it in 1737, and treated with success in 1739, a case of disease in the antrum by injecting it through the natural opening. But, the academy determined that inasmuch as M. Allouel had never published it, Jourdain could not have borrowed it from him, and was therefore entitled to the credit of being the discoverer. It is certain that he was the first to announce it to the world.

The instruments employed for probing and injecting the sinus are, says Bordenave,* 1st, a small silver sound with a button on one end, and a plate in the form of a heart at the other to be held between the fore-finger and thumb of the operator: 2d, a hollow sound without either button or plate, containing a stilet of whalebone, with its extremity extending beyond the sound between the fingers: 3d, a small syringe, with a pipe adapted to the hollow sound. The two first instruments should be curved something like the letter S, and vary a little in size.

The treatment of affections of the maxillary sinus by injections through the nasal opening, having been almost entirely abandoned, a more minute description of the instruments employed for the purpose is not deemed necessary. It may be well, however, before dismissing this part of the subject, to state that the academy, when this method of treatment was proposed by Jourdain, at once appointed commissioners to investigate its merits, and who, after having made a number of trials, came to the conclusion that the introduction of a sound by the nasal opening, although perhaps possible, was so exceedingly difficult, that it could seldom be effected. They attempted it upon each antrum of five subjects, and the result proved that the sound pierced the membranes between the turbinated bones more frequently than it entered the sinus by the natural opening. Their report was therefore unfavourable, and Bordenave in remarking upon this method of gaining access to the cavity, states that while the membranes between the ethmoidal and inferior turbinated bones may be pierced without causing serious injury, it induces us when it happens, to suppose that we have entered the sinus by the natural opening, which "goes to prove that the operation is as difficult as it is uncertain." He adds, however, that while there are cases in which

* Vide Mem. de l'Acad. de Chirurg. 12mo. vol. 12, p. 47.

the use of injections through the natural opening will suffice to effect a cure, these would succeed in only a very small number of the cases, inasmuch as the diseases result more frequently from morbid conditions of the teeth than from any other causes.*

The only advantage then, as is justly remarked by the last named author, that is derived from injections, is the cleansing of the membrane of the antrum, or the disgorgement of the cavity, and this, while the cause remains, will not suffice to effect a cure, while the removal of that, and the giving of vent to purulent or accumulated fluids will of itself, in most instances, be all that is required to bring about a healthy action. The cure, no doubt, will many times be greatly facilitated by the employment of suitable injections, but that these exercise as great a curative influence as many imagine, I have yet to be convinced. They may in those cases where a morbid action has been kept up so long in the mucous membrane of this cavity, as to have nearly destroyed its power to re-act, be highly serviceable, but the difficulty of doing this through the natural opening, as is shown by the result of the experiments of the commissioners appointed by the French Academy, and those of others, who have attempted it, is such as must forever preclude their introduction in that way.

Moreover, M. Allouel and Jourdain, who have attempted to establish the efficacy of injections, by the citation of cases, seem to have overlooked the agency which the removal of the causes, during the employment of the injections, had in bringing about the cure; so that arguments advanced by them in favour of their method of treatment do not prove any thing in its favour. "They might," as Bordenave justly observes, "just as well have been cured without as with them."† Boyer, in alluding to the method proposed by Allouel and Jourdain, asserts that it is opposed both to reason and experience.‡ It is also condemned by almost every writer upon the diseases of this cavity.

When the natural opening is closed, the first indication, as has been stated, is the evacuation of the matter and for this purpose, a perforation should be made into the sinus, and the most proper place for effecting this, it has been shown, is through the alveolar

* Vide, Mem. de l'Acad. Royale de Chirurg. vol. xii. 12mo. p. 51.

† Mem. de l'Acad. de Chirurg. vol. xii. p. 52.

‡ Maladies Chirurgicale, vol. vi. p. 149.

cavity of the second molaris.* It may however be penetrated from that of either of the other molares or bicuspides. The perforation, after the extraction of the tooth, is made with a strait trocar, which will be found much more convenient than those usually employed for the purpose. The point of the instrument after having been introduced into the alveolus, through which it is intended to make the opening, should be pressed against its bottom in the direction towards the centre of the antrum. With the handle of the instrument in the hand of the operator, a few rotary motions will suffice to pierce the intervening plate of bone. If the first opening be not sufficiently large, its dimensions may be increased to the necessary size, by means of a spear-pointed instrument. In introducing the trocar, care should be taken to prevent a too sudden entrance of the instrument into the cavity. Without this precaution, it might be suddenly forced into it and against its opposite wall. The entrance of it is usually attended with a momentary but severe pain, and its withdrawal followed by a sudden gush of fetid mucus.

It is not always necessary to perforate the floor of the antrum after the extraction of a tooth; it occasionally happens, as has already been remarked, that some of the alveolar cavities communicate with it.

An opening having thus been effected, it should be prevented from closing, until a healthy action shall have been established in the lining membrane, and for this purpose a bougie or leaden or silver canula may be inserted into the opening and secured in the manner as previously noticed, to one of the teeth. It should, however, be removed for the evacuation of the secretions of the antrum at least twice a day. The establishing of an opening at the base or most dependent part of the sinus, will, in those cases where a fistula has been previously formed, in most instances, be followed by its speedy restoration. Having proceeded thus far, the cure will be aided by the employment of such general remedies as may be indicated by the state of the constitutional health, and for the reduction of the local inflammation, leeches to the gums and cheek will be found very serviceable. The antrum should, in the meantime, be injected with, at first, some

* Vide, Anat. Phys. and Diseases of the Teeth, p. 261..

mild or bland fluid, and afterwards with gently stimulating liquids.* Diluted port wine, a weak solution of the sulp. zinc, and rose water and also that of copper and rose water,† have been recommended. Diluted tinct. of myrrh may sometimes be advantageously employed, and when the membrane is ulcerated, a weak solution of the nitras argentum‡ will be highly serviceable. For correcting the fetor of the secretions, a weak solution of the chloride of soda or lime, may be injected into the antrum.

Dr. Isaac I. Greenwood, an eminent dentist of New York, employed with success in a case of muco-purulent secretion of the antrum, caused by an alveolar abscess, "suds made from tepid soft water and old Castile soap," and he mentions another, treated in the same way by his father, the late Mr. John Greenwood.§

In cases of muco-purulent secretion simply, a weak decoction of galls,‖ may be injected into the sinus with very considerable advantage.

Injections of a too stimulating nature are sometimes employed. This should be carefully guarded against, by making them at first very weak, and afterwards increasing their strength as occasion may require; but when symptoms of a violent character are in this way produced, they should be combatted by leeches to the gums and fomentations to the cheek.

But, dependent as these affections in most instances are, upon local irritants, greater reliance is to be placed on their removal, and the giving vent to the acrid puriform fluids in the sinus, than

* Anat. Phys. and Diseases of the Teeth, p. 262.

† The following are the formula of Mr. Thomas Bell.

℞. Zinci Sulphat, grs. vi.

Aqua Rosæ, f. ℥ vi. M.

R. Cupri Sulphat, gr. iv.
Aqua Rosæ, f. ℥ vi. M.

In addition to the above he recommends the subjoined.

℞. Tinct. Myrrh, ʒ i.

Decoct. Hordei, f. ℥ vi. M.

‡ This should at first be used very weak, say in the proportion of one grain of Nit. Arg. to two ounces of soft water. Its strength, however, may, if necessary, be gradually increased.

§ Vide American Journal of Dental Science, vol. 2, p. 179.

‖ ℞. Gallæ Pulv. ʒ ii.

Aqua Font. f. ℥ vi. M.

to any therapeutical effects exerted upon the cavity by injections. As adjuvants, they are serviceable, but a cure cannot be accomplished by them, while the exciting cause remains unremoved. This opinion is sustained by the result of the treatment in the two following cases.

CASE IV.—Mr. W. S——, æt. twenty-four years, of a full habit, and slightly disposed to scorbutus, applied to me in the spring of 1839, to obtain my opinion with regard to an affection of his left antrum. It had existed for nearly a year, and the floor of the sinus had been perforated through the alveolus of the second molaris, which had been previously extracted. Injections, first of diluted tinct. of myrrh, and afterwards of a solution of the sulphate of zinc, diluted port wine, &c. &c. had been used regularly once or twice a day for eight months; but still, the matter that was discharged whenever the opening was unclosed, was exceedingly fetid. It had a thick muddy appearance, and turned silver black almost immediately on being brought in contact with it.

Before the antrum was perforated from beneath, this fetid matter was discharged from the nose, which at first, had induced the belief, that the affection was ozena. The heavy pain daily felt in the cheek, and the occasional sudden discharges of purulent matter from the left nostril, when laying upon his right side, led the medical attendant to suspect that the disease was seated in the maxillary sinus, and for the purpose of introducing what he conceived to be the proper remedies, perforated its floor through the alveolus of the second molaris, as just stated. The operation was immediately followed by a discharge of purulent and very fetid matter.

The use of injections, although persisted in for so long a time, were not productive of any permanent benefit, and the patient had almost begun to despair of a cure. His teeth had been examined, but as none in the left side of the upper jaw appeared to be affected with caries, they were not suspected as having any agency in the production of the disease. On examining his mouth, however, I at once perceived that although the teeth were free from decay, they were nevertheless coated with tartar. His gums were inflamed and tumefied; their edges around the bicuspides, the first molaris and dens sapientiæ, were ulcerated, and the alveoli so much wasted that the teeth were considerably loosened, and sensitive to the touch.

Such being the condition of his teeth, gums, and alveoli, the cause of the affection in the antrum, was to my mind apparent. That it was attributable to the irritation in the two last, I felt fully convinced, and therefore advised the immediate extraction of the above-mentioned teeth, and to this operation, as a dernier resort, he reluctantly submitted. Four weeks after their removal the secretions of the antrum ceased to be offensive, and the opening through the alveolus of the second molaris was permitted to close. The injections it is true, that had been previously employed, were continued, but as they had not previously to the removal of the teeth, exerted any curative effect upon the affection of the maxillary sinus, it is not fair to presume that they had subsequently; it was therefore evidently to the extraction of these that its restoration was accomplished.

Case V.—In the summer of 1840, Mr. B. of a strumous habit, æt. nineteen, applied to me for advice, concerning an affection of the right maxillary sinus, that had troubled him for several months. He informed me that nearly a year before, he had broken the crown of one of his teeth; (the second bicuspis of the affected side,) and that a few weeks after he was seized with a severe throbbing pain in the root, this gradually extended to his cheek, but in the course of a few days abated in intensity, though the pain had never entirely subsided. About four weeks after the attack, he began occasionally to discharge a glairy and exceedingly fetid mucus from the right nasal cavity. This continued for several weeks when it nearly ceased, and a similar matter was discharged through the root of the tooth that had at first caused the disturbance, which by this time had become funnelled up to its apex, so that a probe could be passed through it into the antrum, from which cavity the matter seemed to come. To prevent the matter from discharging itself continually into his mouth he kept the canal in the root plugged with raw cotton, which he removed two or three times a day to give vent to the accumulations of purulent mucus.

Persuaded that the disease of the antrum had resulted from the inflammation excited in the alveolus of the second bicuspis by the decayed root that was in it, and the abscess that had in consequence formed and discharged itself into the sinus, the indication of cure, was too obvious to be mistaken. It consisted in the removal of this local irritant, and to this operation he readily and at once submitted. The muco-purulent discharge without other treatment, soon ceased, the opening through the alveolus closed in about ten days, and he has since remained well.

In the foregoing case, the cure was effected without the use of injections of any kind, and simply by the removal of the root of one tooth, the second bicuspis, which had caused the disease.

The particulars of the following case are obtained from "Observations of Bordenave on the Diseases of the Maxillary Sinus,"* a paper embodying reports of forty highly interesting cases.

CASE VI.—"In 1756," says our author, "I was consulted by a lady whose right cheek was tumefied. About a month previous she had experienced acute pain under the orbit of the affected side; and she had felt a pulsation and heat in the interior of the sinus, and the maxillary bone was slightly elevated. These signs determined me to propose the extraction of the third molar tooth,† and the perforation of the antrum through the alveolus. The operation was followed by a discharge of purulent matter, the sinus was afterwards injected, the maxilla gradually reduced itself, and a cure was effected in about two months."

Although injections were employed in the above case, it was no doubt to the giving of vent to the matter contained in the antrum that the cure was attributable. As it regards the cause that gave rise to the affection in the first instance, not a single word is said. It might have resulted from inflammation, lighted up in the sockets of one or more teeth, and propagated from thence to the mucous membrane of this cavity, or from inflammation here, produced by some other cause, and a consequent obliteration of the nasal opening.

The following brief statement is taken from the history of a case narrated by Fauchard.‡

CASE VII.—The child of M. Galois, æt. twelve years, whose first right superior molaris was decayed, had a tumour situated anteriorly upon the upper jaw of the same side, extending up to the orbit. M. Fauchard, supposing this tumour, which was about

* Mem. de l'Acad. Royale de Chirurg. vol. xii. obs. 3, p. 10.

† The bicuspides are called by most French writers, molares, and by the "third molar tooth," he means the one which we call the first.

‡ Le Chirurgien Dentiste, tom. i. obsv. 8, p. 438.

the size of a small egg, had been caused by the carious tooth in question, determined on its extraction as the only means of accomplishing a speedy and certain cure, and the result proved his opinion to have been correct. The removal of the tooth was followed by a large quantity of yellow serous matter, which on examination was found to have escaped from the antrum. The tumour disappeared soon after the discharge of the matter, and a complete cure was effected.

Bordenave, in noticing the foregoing case, inclines to doubt that the tumour communicated with the maxillary sinus, for the reason that the matter escaped through the alveolus of the first molaris immediately after its extraction. He, however, admits that the acumen and knowledge of Fauchard, are such as to have prevented deception in the case.* Admitting then the statement to be correct, and surely the circumstance mentioned by Bordenave does not in the least tend to invalidate it, for it is of frequent occurrence, a cure is effected simply by the removal of a decayed tooth, to the irritation produced by which, the disease was undeniably attributable. The two following cases are described at length by the last named author in the "Memoirs de l'Academie Royale de Chirurgie."†

CASE VIII.—A woman in 1731, had the first superior molaris, the crown of which had been destroyed by caries, extracted. Not many days after the operation, she was attacked with a pain in the upper jaw that extended from the maxillary fossæ to the orbit. The pain was so great as to deprive her of rest, but there was no tumefaction of the cheek or gums. An opening through the alveolus into the sinus was discovered, and into which a probe was introduced by a surgeon. The withdrawal of this was followed by a discharge of yellow fetid matter. M. Lamorier, who was afterwards consulted, removed from the opening a tooth that had been thrust into the antrum and prevented the egress of the matter, which by its retention had become purulent. Injections were employed, and a part of which, at the expiration of thirty days, escaped from the nasal opening. A perfect cure was soon after effected.

In this case, the affection of the sinus, was evidently the result of the injury inflicted upon the socket of the first superior molaris

* Vide, Mem. de l'Acad. Royale Chirurg. vol. xii. p. 13.

† Vide, vol. xii. 12mo. observations 5 and 6, pages 12 and 19.

in an attempt at the extraction of the tooth. Inflammation was excited by this, and the presence of a tooth that had been thrust into the antrum, which extended itself to the lining membrane of this cavity, and caused a temporary obliteration of the nasal opening, so that to effect a cure it was necessary to obtain free vent for the retained matter. In restoring a healthy action to the mucous membrane of the cavity, the injections may have been serviceable.

CASE IX.—A girl, æt. twenty-six years, in having a decayed and painful superior dens sapientiæ on the right side extracted, the tooth was broken and all the roots but one were left in their sockets. These caused an abscess to form, and this was followed, for a short time, by a subsidence of the pain; which however soon returned, and a dull heavy sensation was felt in the antrum of the affected side. From thence the pain extended to the eye and ear. The gums at length became tumefied, and the pain less constant; but the patient, although five teeth were in the meantime extracted, remained in this condition for five years. At this time, 1756, M. Beaupreau, who was consulted, found on examination, that the gums where the first tooth had been extracted, had not entirely united and that a small tubercle had formed here from which a fluid of a bad smell and reddish colour was discharging itself. He introduced a probe into the fistulous hole of this tubercle, which after having overcome some obstacle that at first impeded its passage, penetrated the antrum. The opening was enlarged and mercurial water applied to the carious bone, but it soon closed and the pain which had ceased returned. Injections were resorted to. These discharged themselves in part through the nasal opening, and the patient continued in this way until an exfoliation of the bone took place, when a cure was at once effected.

The cause of the disease in this, as in the preceding cases, was alveolo-dental irritation, and a cure, would at once have been accomplished by the removal of the roots of the tooth that had been left in their sockets, as was proven by the fact, that it was not until they were thrown off with their exfoliated alveoli, that it was effected.

In alluding to these and similar cases, Bordenave concludes that there are not many cases where the extraction of teeth simply, will suffice to effect a cure. The inference, to say the least of it, is unfair, for in the case last given, it was to the presence of the roots of a tooth, that had been fractured in an attempt to extract it, and left in their sockets, that the affection was attributable, and we have every reason to believe, that the cure was wholly owing to their removal.

The history of the following exceedingly interesting case, which was communicated to the Faculty of Medicine, by Professor Dubois, is contained in the 8th number of their bulletin, for the year 13, and also in Boyer's works on Surgical Diseases.

CASE X.—Upon a child between seven and eight years old, at the base of the ascending apophysis of the superior maxillary bone, a small hard round tumor of the size of a walnut, was perceived by its parents. About a year after, the child fell upon its face and caused a considerable discharge of matter from its nose, and at the same time bruising the tumor. No other injury was produced, and the tumour did not increase perceptibly in size, from the eighth to the fifteenth year. During the next year however, it sensibly augmented, and from the sixteenth to the eighteenth year, it attained so great a volume that the floor of the orbit was elevated, which caused a diminution in the size of the eye, and restricted the motions of the eyelids. The arch of the palate was depressed and the nasal fossæ almost closed. The nose was forced to the right side at the upper part of the tumour, and there was a considerable elevation beneath the sub-orbital fossæ. The skin below the inferior eyelid was of a violet red colour and very tense. The upper lip was elevated and the gums on the left side protruded beyond those on the other side of the arch. Respiration was painful, and the patient spoke with difficulty. Sleep was laborious and mastication was attended with pain. "In this state," says M. Boyer, "he was seen by M. Dubois, September 1st, 1802, but as he was not able to determine on the proper operation, M. Sabatier, M. Pelletan and himself were called in. It was the opinion of all, that there was a fungus tumour of the antrum, and for the removal of this M. Dubois was requested to make choice of his own method of operating.

A fluctuation was felt behind the upper lip, and this determined M. Dubois to commence the operation by making an incision there. This was followed by a discharge of a large quantity of a glairy lymphatic substance. Through this opening a sound was introduced into the antrum, and to M. Dubois' surprise, this cavity contained no tumor, but upon moving the sound about it struck upon a hard substance, in the most elevated part of the sinus, which, on being removed proved to be a canine tooth. Preparatory, however, to its extraction, two incisors and one molaris were removed and their alveoli cut away. Injections were afterwards employed and the patient was soon restored to health.

It is not necessary to stop to inquire how this tooth got into the antrum; aberrations of this sort in the growth of the teeth are frequently met with, and some precisely similar instances have already been referred to.*

In all the cases which have as yet been noticed, the affection was traceable to local irritation, and in all except this last, it had originated in the alveolar ridge. But, the following case of muco-purulent engorgement may be thought by some to have been occasioned by a different cause. Yet, there are circumstances connected with the history of even this case, that go far to justify the belief, that if the teeth had been in a healthy condition the affection would not have been produced.

CASE XI.—Mr. G. a labourer, æt. about thirty, of a decidedly scorbutic habit, applied, in the spring of 1834, to an eminent medical gentleman of this city, to obtain his advice concerning an affection of the left side of his face, under which he had been labouring for several months. The physician to whom he applied, after having examined the case, came to the conclusion, that it was mucous engorgement of the maxillary sinus, and requested him to call upon me, and have one of his molar teeth extracted and the floor of the antrum through its alveolus pierced. He at the same time desired, that if his opinion in regard to the nature of the disease proved to be correct, I would take charge of the case altogether. On examining his mouth, I discovered that nearly all his teeth of both jaws, gums and alveoli were extensively diseased, and on inquiry, obtained from him the following statement with regard to the commencement and progress of the affection of the antrum.

About six months previous to this time, having been exposed while pursuing his ordinary avocations, to very inclement and several sudden changes of weather, he contracted a severe cold; in consequence of which, he was confined to his bed for several days; during this time, he was twice bled, took two cathartics, and other medicines.

* Vide note, to page 48.

The disease at first concentrated itself in his head, face, and jaws, which at the expiration of eight or ten days, was subdued by the above treatment, with the exception of the pain in his left cheek, and soreness in the superior teeth of the same side. The pain in his cheek, although not constant, still continued; the nasal cavity of that side ceased to be supplied with its usual secretion, the teeth became more sensitive to the touch, and finally at the end of four months, a slight protuberance of the cheek was observable, accompanied by a tumour upon the left side of the palatine arch, which, when I first saw him had attained to half the size of a black walnut, and it was by the fluctuation here felt that the physician whom he first consulted, was induced to suspect the true nature of the disease.

Acting under the direction of the medical gentleman, under whose care the patient had placed himself, I extracted the second left superior molaris, and through its alveolus penetrated the antrum by means of a straight trocar, and after the withdrawal of which, a large quantity of glairy fetid mucous fluid was discharged. The perforation was kept open by means of a bougie, secured with a silk ligature to an adjoining tooth, as recommended by Deschamps, and the antrum injected three times a day; at first, simply with rose water, to which a small quantity of sulphate of zinc was afterwards added. By this treatment, the lining membrane of the antrum at the expiration of five weeks was restored to health, so that the secretions that escaped through the perforation were inodorous.

The patient no longer experiencing any inconvenience, withdrew the bougie, and allowed the aperture to close. In about two months, he again presented himself to me, and was similarly affected as when I first saw him. I now extracted the first superior left molaris, and perforated the antrum through its alveolus, and a quantity of fetid mucous fluid was again discharged; the dens sapientiæ, and the first and second bicuspides of of the affected side, which were carious, were also extracted. Injections of sulphate of zinc and rose water, diluted tincture of myrrh, diluted port wine, a decoction of Gallæ, were alternately employed for three months, at the expiration of which time, the nasal opening which had been previously closed, was re-established, and a perfect cure was effected.

[TO BE CONTINUED.]

